# HHV-6A Infection of Endometrial Epithelial Cells Affects miRNA Expression and Trophoblast Cell Attachment

**DOI:** 10.1007/s43032-019-00102-8

**Published:** 2020-01-01

**Authors:** Daria Bortolotti, Irene Soffritti, Maria D’Accolti, Valentina Gentili, Dario Di Luca, Roberta Rizzo, Elisabetta Caselli

**Affiliations:** Section of Microbiology and Medical Genetics, Department of Medical Sciences, University of Ferrara, via Luigi Borsari 46, 44121 Ferrara, Italy

**Keywords:** HHV-6A, Infection, Endometrium, miRNA, Pregnancy

## Abstract

We recently reported that human herpesvirus 6 (HHV-6) infection is frequently present in endometrial tissue of women with unexplained infertility, and that virus infection induces a profound remodulation of miRNA expression in human cells of different origin. Since specific miRNA patterns have been associated with specific pregnancy outcomes, we aimed to analyze the impact of HHV-6A infection on miRNAs expression and trophoblast receptivity in human endometrial cells. To this purpose, a human endometrial cell line (HEC-1A) was infected with HHV-6A and analyzed for alterations in the expression of miRNAs and for permissiveness to the attachment of a human choriocarcinoma trophoblast cell line (JEG-3). The results showed that HHV-6A infection of endometrial cells up-modulates miR22 (26-fold), miR15 (19.5-fold), and miR196-5p (12.1 fold), that are correlated with implant failure, and down-modulates miR18 (11.4 fold), miR101-3p (4.6 fold), miR181-5p (4.9 fold), miR92 (3.3 fold), and miR1207-5p (3.9 fold), characterized by a low expression in preeclampsia. Moreover, HHV-6A-infected endometrial cells infected resulted less permissive to the attachment of trophoblast cells. In conclusion, collected data suggest that HHV-6A infection could modify miRNA expression pattern and control of trophoblast cell adhesion of endometrial cells, undermining a correct trophoblast cell attachment on endometrial cells.

## Introduction

During embryo implantation, more than 500 miRNAs are produced [1, 2], whose expression varies in response to environmental factors (hypoxia, signaling pathways, epigenetic modification), and during the different stages of implantation [3, 4], contributing to the regulation of placental development, trophoblast cell proliferation, apoptosis, migration, invasion, and angiogenesis.

Based on their critical role, specific miRNAs have been associated with pregnancy outcomes [5].

In the endometrium, miRNAs are involved in the dynamic changes associated with the menstrual cycle, and are implicated in implantation and reproductive disorders. Analysis of miRNAs in female human reproductive tissues has shown that both the uterus (endometrium, myometrium, and cervix) and the ovaries, have high enrichment of individual miRNAs [6–10]. Interestingly, the involvement of miRNAs in altered endometrial receptivity, abnormal pregnancies, endometriosis, gynecological malignancies and fertility disorders has been reported [3]. In subfertility, there is a marked difference in miRNA profiles when compared with the fertile population, supporting the role of miRNAs as major bioregulatory molecules of many physiological processes including reproduction [11].

We recently reported a high rate of human herpesvirus 6A (HHV-6A) infection in endometrial biopsies from women with unexplained infertility [12], and showed that primary human endometrial cells are permissive to HHV-6A infection [13]. Furthermore, we observed that Human Herpesvirus 6 (HHV-6) infection induces a profound remodulation of miRNA expression in human cells of different origin [14, 15].

Based on these observations, here we analyzed the impact of HHV-6A infection on miRNAs expression and trophoblast receptivity in human endometrial cells.

## Materials and Methods

### Cells

The human endometrial HEC-1A cell line (ATCC HTB-112) was used for infection experiments. Cells were propagated in McCoy’s medium (ATCC 30-2007), supplemented with 10% fetal calf serum (FCS) (complete McCoy’s medium).

The human JEG-3 choriocarcinoma cell line (ATCC HTB-36) was used for spheroid assays. Cells were cultured in RPMI 1640 medium (ThermoFisher Scientific, Milan, Italy) supplemented with 10% FCS (complete RPMI medium).

### Virus

Cell-free HHV-6A inocula were obtained by density gradient purification from culture supernatant and cell lysate of HHV-6A infected J-Jhan T cells, as previously described [13, 16, 17]. Virus inocula were maintained in phosphate buffered saline (PBS) with 5% bovine serum albumin (BSA) at − 80 °C until use, and quantified by quantitative real-time PCR (qPCR) targeting U94 virus gene [14]. All the experiments were performed using the same virus inoculum, containing 10^10^ genome copies/ml, corresponding to about 10^9^ infecting particles/ml, as previously described [17]. UV-inactivated virus inocula were obtained as previously described [18].All experiments involving virus production and infection were performed under the standard BLS-2 biosafety level.

### Endometrial Cell Infection

HEC-1A cells were seeded in 6-well plates (5 × 10^5^ cells/well) 24 h before infection, to obtain optimal density, and infected with gradient-purified cell-free HHV-6A at a 10:1 multiplicity of infection (MOI, virus genomes:cell ratio), as previously described [17]. Virus adsorption was carried out in 2% FCS medium for 3 h; then, cells were washed with PBS to eliminate unbound virus, and fresh complete McCoy’s medium was added. Control cells were uninfected or treated with UV-inactivated virus inoculum. At 1, 4, 8, 14, and 21 days post infection (d.p.i.), aliquots of cell cultures were collected and analyzed for virus DNA presence and transcription, and for expression of miRNAs. Evaluation of cell viability was performed by cell counting after Trypan Blue exclusion test.

### Analysis of Virus Replication

Virus presence and transcription in HEC-1A cells were evaluated by analyzing DNA and RNA from infected cells. Briefly, 100 ng of total extracted DNA was analyzed by specific qPCR targeting U94 gene, and 200 ng of total extracted RNA was retrotranscribed using the RT2 First Strand kit (Qiagen, Hilden, Germany) and analyzed by two qPCRs targeting U94 and U42 viral genes, as previously described [14, 16]. The human RNaseP housekeeping gene was amplified as a control. In addition, viral antigen production was analyzed by immunofluorescence, using an anti-HHV-6 p41-PE IgG (SantaCruz Biotechnology, Heidelberg, Germany) and Hoechst (ThermoFisher Scientific, Milan, Italy) for nucleus staining. Images were obtained with Nikon Eclipse TE2000S, equipped with a digital camera.

### miRNA Analyses

For miRNA analyses, total extracted RNA was retrotranscribed using the miScript RT kit (Qiagen, Hilden, Germany), and 100 ng of obtained cDNA were analyzed by the “Human Inflammatory Response & Autoimmunity” microarray (Qiagen, Hilden, Germany), able to detect and quantify 84 different miRNAs simultaneously. In addition, 29 further individual assays were performed, including miRNAs specifically involved in pregnancy and not included in the array: miR18, miR21-5p, miR22, miR24, miR30b-5p, miR31, miR92, miR125-3p, miR125b-5p, miR145-5p, miR155_1, miR155-2, miR196b-5p, miR199b-5p, miR200_1, miR200_2, miR222, miR374a-5p, miR378a-3p, miR422, miR423-5p, miR424-5p, miR449a, miR517, miR572, miR575, miR1207-5p, miR3663-3p, miR4306 and miR5739. miRTC_1, and SNORD11 were used as controls (Qiagen, Hilden, Germany). Amplification results were analyzed and normalized by a specific Qiagen software, to obtain comparable values between control and infected cells at each time post infection.

### Trophoblast Attachment Assay

Based on preliminary experiments set up to optimize the kinetics of JEG-3 spheroid growth, JEG-3 cells at 80% confluence were seeded at 3 × 10^4^ cells/ml in complete RPMI medium additioned with 1.5% agarose [19], allowing to obtain spheroids of 100–250 μm in diameter at 2–4 days of culture. JEG-3 spheroid size was measured using an optic microscope equipped with a calibrated eyepiece reticule. Cell viability was measured by DAPI stain.

For attachment assays, HEC-1A cells were seeded in 12-well plates (Nunc), at 1.6 × 10^6^ per ml/well, and cultured for 2 days. Then, JEG-3 spheroids were transferred onto HEC-1A monolayers one by one with a fine Pasteur pipette. After 0.5, 1, and 2 h of incubation, the attachment rate was calculated by the NucSpot Live 488 test (Biotium, Fremont, CA, USA), under fluorescence microscopy, and expressed as the rate between attached and seeded spheroids.

### Statistical Analysis

Statistical analysis was performed by Student’s *t* test for comparison between infected and control cells, with Bonferroni correction for multiple comparisons (microarray data). Linear regression analysis was conducted to evaluate the time dependence of the sphere attachment rate. A corrected *p* (*p*_*c*_) value ≤ 0.05 was considered significant.

## Results

### HHV-6A Infection of Endometrial Cells

**Fig. 1:**
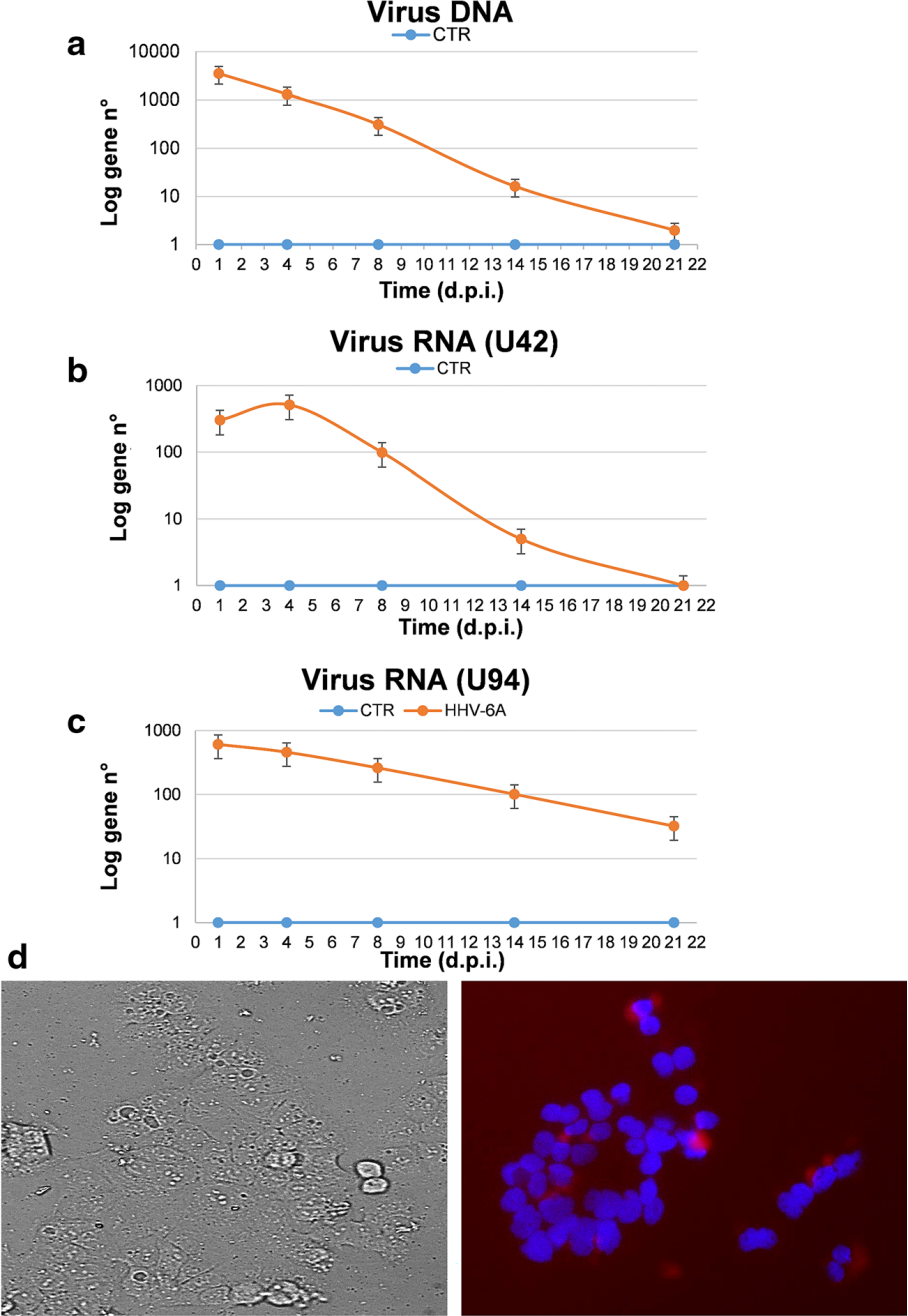
HHV-6A infection in endometrial cells. HEC-1A cells were seeded at optimal density and infected with HHV-6A (m.o.i. 10:1). Control cells (CTR) were uninfected. After 1, 4, 8, 14, and 21 days post infection (d.p.i.) total DNA or RNA were extracted from collected cells. **a** Virus DNA presence was analyzed on total extracted DNA, by a qPCR targeting virus U94 gene. **b**, **c** Virus transcription was evaluated on total extracted RNA from collected cells, by qPCRs targeting U94 andU42 genes. **d** HEC-1A cell line infected with HHV-6A was stained with IgG anti-HHV-6 p41-PE; nuclei were stained with Hoechst. Images were taken by Nikon Eclipse TE2000S, equipped with a digital camera. Left panel bright field, right panel fluorescence field. Original magnification × 40.The results are expressed as Log gene copy number, and represent the mean value ± SD of duplicate samples in three independent experiments

Human endometrial HEC-1A cells were permissive to HHV-6A infection, as shown by analysis of virus replication at 1, 4, 8, 14, and 21 days post infection (d.p.i.) (Fig. 1). Cell viability was not affected by virus infection till the end of the experiment (21 d.p.i.), as judged by Trypan blue exclusion assay (not shown). Efficiency of infection was about 45%, similar to what was previously observed with the KLE endometrial epithelial cell line [13]. Virus DNA (quantified by U94 qPCR) was detectable until the end of the experiment, though it decreased over time, resulting in fewer than 10 copies per 100 ng of cellular DNA (corresponding to about 10^4^ cells) at 21 d.p.i. (Fig. 1a). Analysis of virus transcription confirmed that HHV-6A was actively transcribing lytic genes (U42) (Fig. 1b) and expressing the lytic virus antigen p41 till 8 d.p.i. (Fig. 1d); afterwards, virus transcription decreased and only low levels of U94, which is also expressed during virus latency, were detectable (Fig. 1c).

### HHV-6A Impact on miRNA Expression in Endometrial Cells

**Fig. 2:**
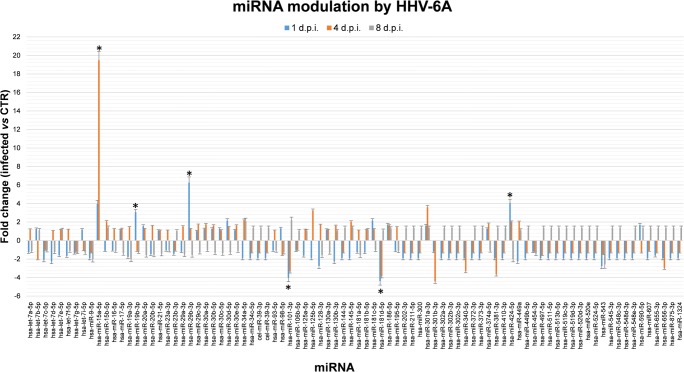
miRNA modulation by HHV-6A infection in endometrial cells: microarray results. HEC-1A cells were seeded at optimal density and infected with HHV-6A (m.o.i. 10:1). Control cells (CTR) were uninfected. After 1, 4, and 8 days post infection (d.p.i.) RNA including miRNA fraction was extracted from collected cells, and analyzed by a microarray evidencing simultaneously 84 miRNAs. Results are expressed as fold change values (infected vs control) and represent mean ± SD of duplicate samples in two independent experiments. Asterisks indicate statistically significant values (*p*_*c*_ < 0.05)

The analysis of miRNA expression in infected endometrial cells showed that HHV-6A had a significant impact on miRNA expression; by contrast, no alterations were observed in cells infected with UV-inactivated virus, where miRNA levels were superimposable with those detected in uninfected endometrial cells. In particular, at 1 d.p.i., there was a significant up-modulation of miR15a-5p (4.0-folds), miR19b-3p (3.1-folds), miR29b-3p (6.3-folds), and miR424-5p (4.1-folds), and down-modulated miR101-3p (− 4.1-folds) and miR181d-5p (− 4.4-folds) (*p*_*c*_ ≤ 0.05, as detected by Student’s *t* test with Bonferroni correction for multiple comparison) (Fig. 2). At 4 d.p.i., the up-modulation of miR-15a-5p was more evident (19.5-folds), while that of miR424-5p was detectable but less abundant (2.2-folds); the down-modulation of miR101-3p (− 3.5-folds) and miR181d-5p (− 3.8-folds) was maintained, whereas the expression of the other miRNAs modified at 1 d.p.i. was transient. At 8 d.p.i., no substantial variation was observed, as the few differently expressed miRNAs showed only twofold variations compared to controls.

**Fig. 3:**
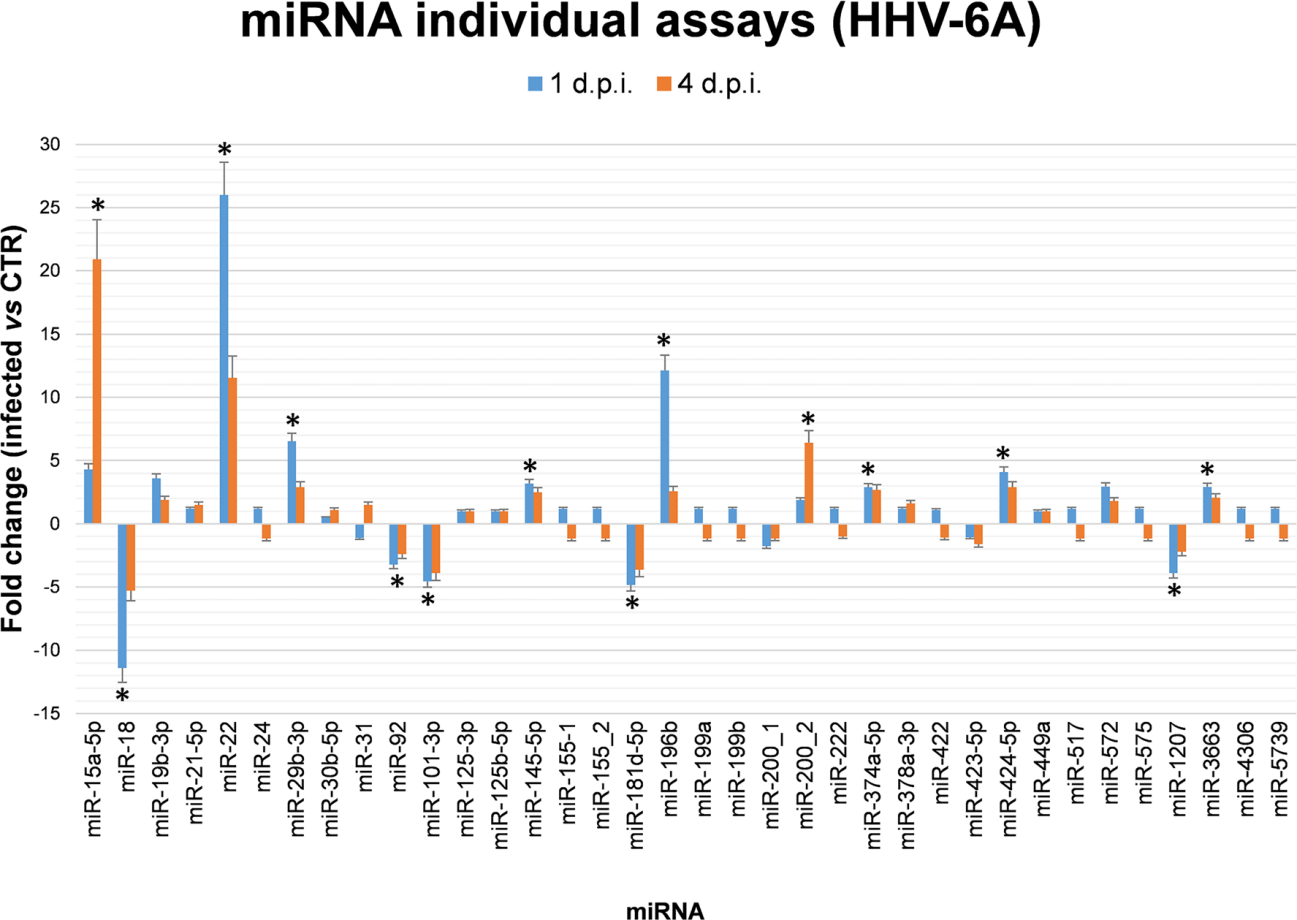
HHV-6A modulation of individual miRNAs associated with pregnancy. HEC-1A cells were seeded and infected as described for microarray analyses. Total RNA extracted from cell samples at 1 and 4 days post infection (d.p.i.) were analyzed by individual qPCR assays targeting the indicated miRNAs. Results are expressed as fold change values (infected vs control) and represent mean ± SD of triplicate samples in two independent experiments. Asterisks indicate statistically significant values (*p*_*c*_ < 0.05)

Furthermore, individual assays showed that other 10 miRNAs were affected by HHV-6A infection. Namely, miR18, miR92, and miR1207-5p were down-modulated (11.4-, 3.3-, and 3.9-folds respectively), several miRNAs (miR145-5p, miR200_2, miR374a-5p, miR424-5p, miR3663-3p) were over-expressed (from 2.1- to 6.4-folds), and miR22 and miR196b-5p were highly expressed (25- and 12-folds, respectively) (Fig. 3). Associated functions of analyzed miRNAs in pregnancy and the impact of HHV-6A infection on their expression are summarized in Table 1.

**Table 1:** Function of tested miRNA and HHV-6A effect on their expression

miRNA	Modulation	Condition	Reference	HHV-6A infection (folds*)
miR-15-a-5p	↑	IF	Yang et al. 2016	↑ 19.5
miR-18	↓	IF, PE	Liu et al. 2016	↓ 11.4
miR-19b-3p	↓	PE, EPL	Ventura et al. 2013	↑ 3.6
miR-21-5p	↑	RIF	Shi et al. 2017	Not modulated
miR-22	↑	RIF	Ma et al. 2015	↑ 26.0
miR-24	↓	IF	Liu et al. 2016	Not modulated
miR-29b-3p	↑	Angiogenesis inhibition	Fang et al. 2011 Jiang et al. 2017	↑ 6.3
miR-30b-5p	↑	RIF	Shi et al. 2017	Not modulated
miR-31	↓	IF	Kresowik et al. 2014	Not modulated
miR-92	↓	IF	Liu et al. 2016	↓ 3.3
miR-101-3p	↓	Trophoblast apoptosis	Zou et al. 2014	↓ 4.6
miR-125-3p	↑	EPL	Hosseini et al. 2018	Not modulated
miR-125b-5p	↑	RIF	Shi et al. 2017	Not modulated
miR-145-5p	↑	RIF	Shi et al. 2017	↑ 2.5
miR-155_1	↓	RPL	Al-Shorafa et al. 2012	Not modulated
miR-155_2	↓	RPL	Al-Shorafa et al. 2012	Not modulated
miR181d-5p	↓	Recurrent miscarriage	Zhao et al. 2017	↓ 4.9
miR-196b-5p	↑	RIF	Shi et al. 2017	↑ 12.1
miR-199a-5p	↑	RIF	Shi et al. 2017	Not modulated
miR-199b-5p	↑	RIF	Shi et al., 2017	Not modulated
miR-200_1	↑	IF	Haraguchi et al. 2014	Not modulated
miR-200_2	↑	IF	Haraguchi et al. 2014	↑ 6.4
miR-222	↓	RPL	Al-Shorafa et al. 2012	Not modulated
miR -374a-5p	↑	RIF	Shi et al. 2017	↑ 2.9
miR-378a-3p	↓	EPL	Hosseini et al. 2018	Not modulated
miR-422	↓	PE	Gunel et al. 2017	Not modulated
miR-423-5p	↑	EPL	Hosseini et al. 2018	Not modulated
miR-424-5p	↑	RIF	Shi et al. 2017	↑ 4.1
miR-449a	↑	RIF	Shi et al. 2017	Not modulated
miR-517	↓	EPL	Hosseini et al. 2018	Not modulated
miR-572	↓	RIF	Shi et al. 2017	Not modulated
miR-575	↑	EPL	Hosseini et al. 2018	Not modulated
miR-1207-5p	↓	RIF	Shi et al. 2017	↓ 3.9
miR-3663-3p	↑	EPL	Hosseini et al. 2018	↑ 2.1
miR-4306	↓	RIF	Shi et al. 2017	Not modulated
miR-5739	↓	RIF	Shi et al. 2017	Not modulated

*Results represent mean values of triplicate sample in two independent experiments, and are expressed as fold change (infected versus control uninfected cells); the highest value of HHV-6A-induced modulation is reported. RIF, recurrent implantation failure; IF, implantation failure; EPL, early pregnancy loss; RPL, recurrent pregnancy loss; PE, preeclampsia

### Impact of HHV-6A on Endometrial Permissiveness to Trophoblast Invasion

**Fig. 4:**
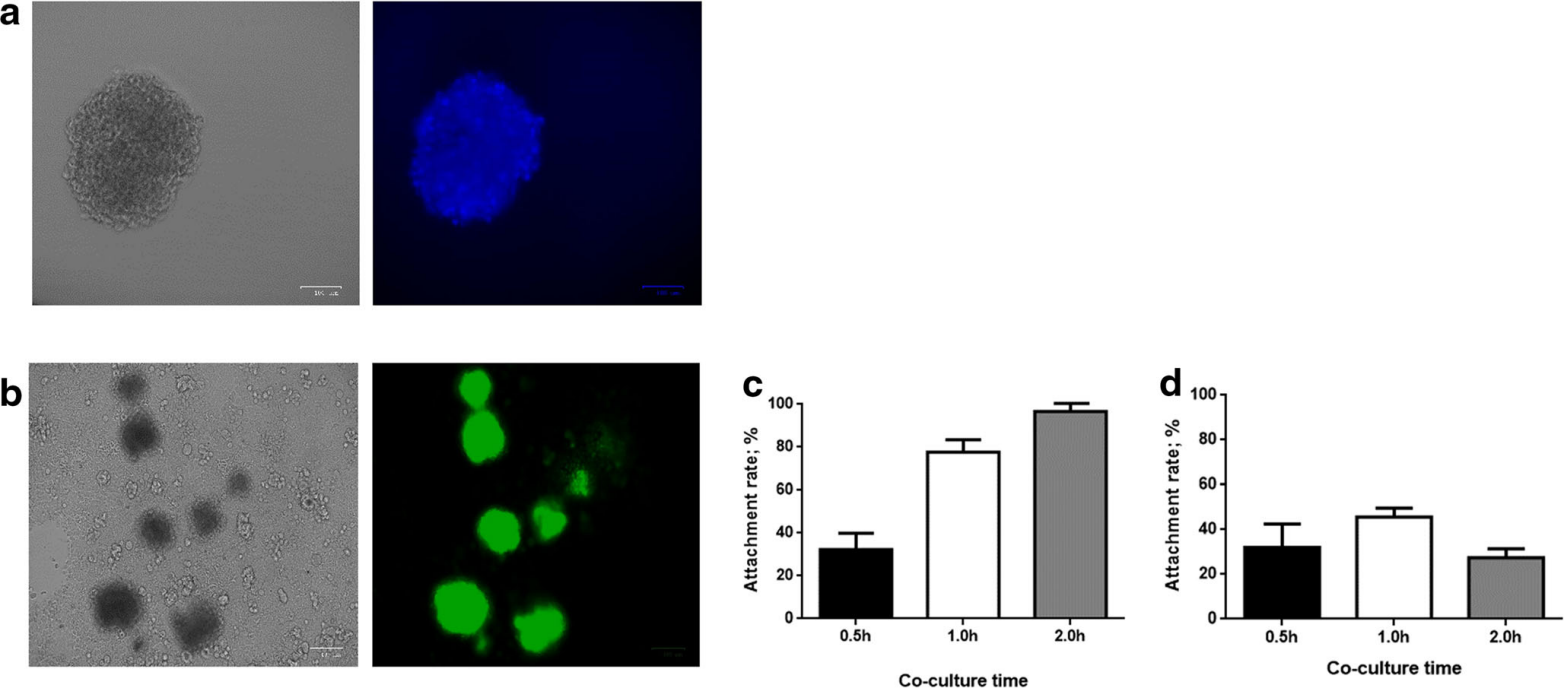
Attachment of JEG-3 spheroids to endometrial cell monolayers. **a** Viability of JEG-3 spheroids of 100–250 μm in diameter, grown for 2–4 days in culture, measured by DAPI staining: representative picture of a 4-days JEG-3 spheroid; optical microscope, original magnification × 40. **b** Attachment of JEG-3 spheroids, stained with Syto9 (ThermoFisher; Milano, Italy), to HEC-1A endometrial cell monolayers; optical microscope, original magnification × 40. **c** Graphical representation of the attachment kinetics, calculated by the NucSpot Live 488 test (Biotium, CA, USA), under fluorescence microscopy, of JEG-3 spheroids to uninfected HEC-1A monolayers. **d** Graphical representation of the attachment kinetics of JEG-3 spheroids to HHV-6A-infected HEC-1A cells. Results are expressed as mean ± SD of triplicate samples in two independent experiments

To evaluate the effect of HHV-6A infection on endometrial permissiveness to trophoblast invasion, extravillous throphoblast JEG-3 cells were grown in complete RPMI medium additioned with agarose to form multicellular spheroids. Then, to examine trophoblast-endometrial cell interactions, JEG-3 spheroids were co-cultured with infected or uninfected human endometrial HEC-1A cells. Briefly, JEG-3 spheroids grown for 2–4 days in culture, with viability > 99% (Fig. 4), were seeded on HEC-1A monolayers to perform an attachment assay. The results showed that the attachment rate of JEG-3 spheroids to uninfected endometrial monolayers gradually and significantly increased over time (linear regression analysis, *p* < 0.001), reaching 79.5% after 1 h and 100% after 2 h of co-culture. By contrast, the attachment of JEG-3 spheroids was impaired when endometrial cell were HHV-6A infected (linear regression analysis, *p* = 0.0025), being 50% compared with controls after 1 h (*p* = 0.02), and 35% compared with controls after 2 h of co-culture.

## Discussion

The results show that HHV-6A infection has a profound impact on miRNA expression in human endometrial cells, modulating at least 16 miRNAs having potentially critical roles during embryo implantation. All of the most upregulated miRNAs (miR22, miR15, and miR196-5p) have been correlated with implant failure in the literature.

In particular, miR22 was associated with repeated implant failure (RIF) [20]. miR15 is the prototype of the “miR15 family,” a large conserved family that plays important roles in vascular development and associated diseases: it is upregulated during inflammation and is associated with the inhibition of trophoblast cell invasion and neo-angiogenesis in the placenta [21]. miR196-5p was found to be over-expressed in women with RIF and is considered part of the microRNA signature defining the outcome of implantation during the implantation window [5].

HHV-6A infection of endometrial cells also induced up-regulation of miR424-5p, another member of the “miR15 family” that is known to play a unique role in the placentation process [22], and that is up-modulated in women with RIF [5]; of miR29, a member of the “apoptomir” family, suppressing angiogenesis and invasion in tumors [23] and involved in arterial calcification [24], suggesting that during implantation it might interfere with a correct placental neo-angiogenesis; of miR21-5p, miR145-5p, miR374a-5p, and miR3663-3p, all associated with RIF or early pregnancy loss (EPL) [5, 25], and of miR19b-3p, a member of the miR17-92 cluster downregulated in villous samples from women with EPL [26].

On the other hand, HHV-6A infection induced in endometrial cells the down-modulation of miR18, miR101-3p, miR181-5p, miR92, and miR1207-5p. Notably, miR18 expression was found to be decreased in circulating blood and placentas of women suffering from preeclampsia (PE) [27, 28]; miR101-3p reduction was associated with trophoblast apoptosis [29] in PE placentas; decreased miR181-5p was reported in women with recurrent miscarriage [30], although other works reported a possible association between increased expression of miR181 and abnormal pregnancy, due to its role in attenuating the immunosuppressive properties of mesenchymal stem cells [31]; miR92, belonging to the miR17-92 cluster normally upregulated during implantation in receptive uteri [4], was reported to be downregulated in EPL placentas [26, 32], and miR1207-5p down-regulation was reported in women with RIF compared with controls [5].

To support the hypothesis of a role of HHV-6A infection in implant failure, as an initial proof of concept, we also showed that HHV-6A infected endometrial cells are less permissive to the attachment of trophoblast cells, suggesting a direct effect of endometrial infection on the trophoblast attachment rate. Based on our observations, it might be hypothesized that virus-induced alterations in the endometrial cells, such as miRNA expression, and/or in the cell microenvironment, controlling trophoblast cell behavior, might be associated with HHV-6A interference with correct implantation. Further researches will however be needed, to confirm the results in primary human endometrial cells, as well as with other trophoblast-derived spheroids, as JEG-3 cells are not extravillous cytotrophoblasts but are choriocarcinoma cells with trophoblast-like feature. In addition, future studies would be important to test miRNA-specific inhibitors/vectors, in order to demonstrate the implication of the individual miRNA modulated in HHV-6A-infected endometrial cells, thus clarifying the mechanisms by which HHV-6A might interfere with the correct recognition between endometrial cells and trophoblast cells, undermining correct embryo implantation.
